# Developments in the Fermentation Process and Quality Improvement Strategies for Mead Production

**DOI:** 10.3390/molecules190812577

**Published:** 2014-08-19

**Authors:** Antonio Iglesias, Ananias Pascoal, Altino Branco Choupina, Carlos Alfredo Carvalho, Xesús Feás, Leticia M. Estevinho

**Affiliations:** 1 Department of Anatomy and Animal Production, Faculty of Veterinary Science, University of Santiago de Compostela, Lugo, Galicia E-27002, Spain; E-Mail: antonio.iglesias@usc.es; 2 CIMO-Mountain Research Center, Agricultural College of Bragança, Polytechnic Institute of Bragança, Campus Santa Apolónia, Bragança E 5301-855, Portugal; E-Mails: ananiaspascoal@hotmail.com (A.P.); albracho@ipb.pt (A.B.C.); leticia@ipb.pt (L.M.E.); 3 Grupo de Pesquisa Insecta, Centro de Ciências Agrárias, Ambientais e Biológicas, Núcleo de Estudo dos Insetos, Universidade Federal do Recôncavo da Bahia, Cruz das Almas BA E 44380-000, Brazil; E-Mail: carlos.alfredo@gmail.com; 4 Department of Organic Chemistry, Faculty of Sciences, University of Santiago de Compostela, Lugo E-27080, Spain

**Keywords:** mead production, fermentative microorganisms, fermentation progress, quality procedures, honey

## Abstract

Mead is a traditional alcoholic drink derived from the fermentation of diluted honey in the presence of appropriate yeast. Its modern production, in general terms, involves the addition of nutrients to initial diluted honey, pasteurization, yeast inoculation, fermentation and removal of impurities. Undesirable events along the process have been reported; among them, we highlight: delayed or arrested fermentations, modified and unpleasant sensory and quality parameters of the final product. These problems have been linked to the inability of yeasts to accomplish their role in extreme growth conditions. Emphasis has also been placed on the long fermentation times required, ranging from weeks to months, particularly when traditional procedures are applied and when the honey concentration is low. A series of alterations to the must and technological changes have been proposed in order to optimize the mead production process. In this context, this review examines the evidence that aims to improve meads’ quality and make the production process easier and more efficient, by clarifying the source of unexpected events, describing the implementation of different fermentative microorganisms and using new methodologies.

## 1. Introduction

Given that honey production is a significant economic activity in Europe, the development of honey-derived products appears to be a sound alternative to provide innovative alcoholic drinks to the consumers and to increase the profit of the beekeeping industry.

From the fermentation of honey alcoholic beverages can be obtained many, among which: sherry type wine, sparkling wine, fruit-honey wine and different types of mead. These products have different flavors depending on the floral source of the honey, the yeast used in the fermentation and the presence of additives [1].

Mead is a traditional alcoholic beverage containing from 8% to 18% of ethanol (v/v) produced by the fermentation of a diluted solution of honey [2,3]. Despite the fact that traditional mead is simply a fermented mixture of honey and water, many variations have existed throughout the ages, ranging from the traditional to complex mixes of fruit juices and spices. Indeed, different types of mead have been reported. The most common are metheglin (mead containing spices or herbs), melomel (mead with fruit juices), hippocras (pyment with herbs and spices) and sack mead (produced with superior concentration of honey) [4].

Fructose is generally the most abundant simple sugar found in honey and the mead fermentation process is longer than most alcoholic fermentations, where other sugars are present and in higher concentrations [5]. In fact, this fermentation often takes several months to complete, depending on the type of honey, yeast strain and honey-must composition [6]. During this process some problems usually occur, due to the inability of yeast strains to respond and adapt to unfavorable stressful growth conditions found in honey [2]. Consequently, complications such as a lack of uniformity of the final product arise, probably due to the variability of honey composition and to the occurrence of re-fermentations by yeasts or acetic and lactic bacteria, which may increase volatile acidity and promote abnormal ester production, affecting the sensory qualities of the final product [7].

Even though mead is perhaps the oldest fermented drink in the world, produced mainly in an empirical way, its production has suffered in recent years, partially due to the lack of scientific progress in this field [2]. Even though there is not much scientific information regarding honey-must fermentations, it is generally accepted that the improvement of meads’ quality includes the development of adequate additive formulations and the optimization of the fermentation conditions [8]. As such, this review aims to analyze the evidence regarding yeast selection, production methodologies and strategies for quality improvement in mead production.

## 2. Background of Mead Production

References to mead in the literature and archeological findings have been found dating back nearly 3000 years [9]. The origins of this beverage can be traced back to African countries. It was later produced throughout the Mediterranean basin and Europe, playing an important role in the early ancient civilizations. In fact, mead was an important part of the rituals of the Celts, Anglo-Saxons and Vikings, referred to as the drink of nobles and gods, providing immortality and knowledge [4] and believed to have magical and healing powers even capable of increasing strength, virility and fertility. Lucius Junius Moderatus Columella in his *De Re Rustica* (42 CE) and Pliny the elder in his *Naturalis Historia* of (77 CE), reported the empirical use of honey for the production of mead and provided a detailed description of the procedure used for obtaining the traditional beverage.

Fermented drinks obtained from honey, among which mead, are though to be the oldest alcoholic beverages known to man, made thousands of years before either wine or beer were produced. Evidence regarding the collection of honey dates back to at least 8000 BCE and is thought to date back into the Paleolithic period (*i.e*., pre-10,000 BCE).

It is important to mention that the production of mead in the southern Europe declined when the grapes were discovered as a less expensive and more predictable source for the production of alcoholic beverages. Despite this, in the northern countries, where these fruits were less available, the popularity of mead persisted [10].

The scientific advances conducted on mead include the development of additive formulations and fermentation conditions [10,11,12,13,14] and processing improvements via ultrafiltration and flash pasteurization [15]. Concerning cells’ immobilization process, it was first applied to this beverage in the 1980s [16].

Actually, mead seems to be a good option for increasing the income of honey producers, allowing the development of a beverage little known in some countries but possessing great commercial potential. This is also in line with the present situation of consumers demanding more options and a willingness to try new products. Indeed, the great potential of mead is already evident in some countries, for example in the United States, were there are currently more than 45 commercial meaderies, a number that is continuing to increase [17].

## 3. Yeasts Used in Mead Production

Yeasts used in mead production are starter yeasts, which metabolize sugars, such as glucose and fructose, resulting in the formation of ethanol and carbon dioxide [18]. Yeast strain selection plays a key role since it influences the efficiency of conversion from sugar to alcohol.

In the first studies regarding yeast selection for mead production it was suggested that, due to the low homology between honey and wine must regarding sugars and nitrogen concentration, *Saccharomyces cerevisiae* could not be the most suitable for mead production [19,20,21]. However, studies conducted using many strains of *S. cerevisiae*, among which C11-3 [6], BRL-7 [22] and UCD522 [8] from culture collections, as well as commercial strains, such as Premier cru^®^ [2], ENSIS-LE5^®^ [1], Fermol Reims Champagne^®^ and ICV^®^ D47 [23], concluded that this yeast, similar to the used in the production of wine, beer, and champagne is adequate for mead production.

Recently, the fermentative performance of selected strains isolated from honey and wine [2,21] and commercial yeasts starter cultures has been studied [24]. However, to date, yeasts isolated from honey have not shown advantages over easily obtained commercial strains, despite the identified resistance to ethanol, sulphurous oxide and high concentrations of sugars [2]. As such, due to the advantages related to yeast stability and the easier access for the mead-makers, the inoculation of the honey-must with commercial yeasts used in the production of white wines is recommended [23]. According to Pereira *et al.*, the most appropriate yeast strain is D47 ICV because it shows a high fermentation rate and a low production of volatile acidity and acetaldehyde [3]. Acetaldehyde is a reactive, flavor active compound, which has been suspected to cause long-term adverse effects in consumers [25].

However, despite the use of starter cultures for mead production several problems still persist [2], what could be due to the use of yeast strains that are not suitable for the specific composition of the honey-must or associated with the stress conditions found, among which high osmocity, low concentration of essential nutrients [26], low mineral content, low pH [27] and low buffer capacity [8].

Therefore, it will be necessary to find and isolate yeast strains with higher resistance and better fermentation performance under the harsh conditions of mead production [7].

## 4. Steps Involved on Mead Production

**Figure 1:**
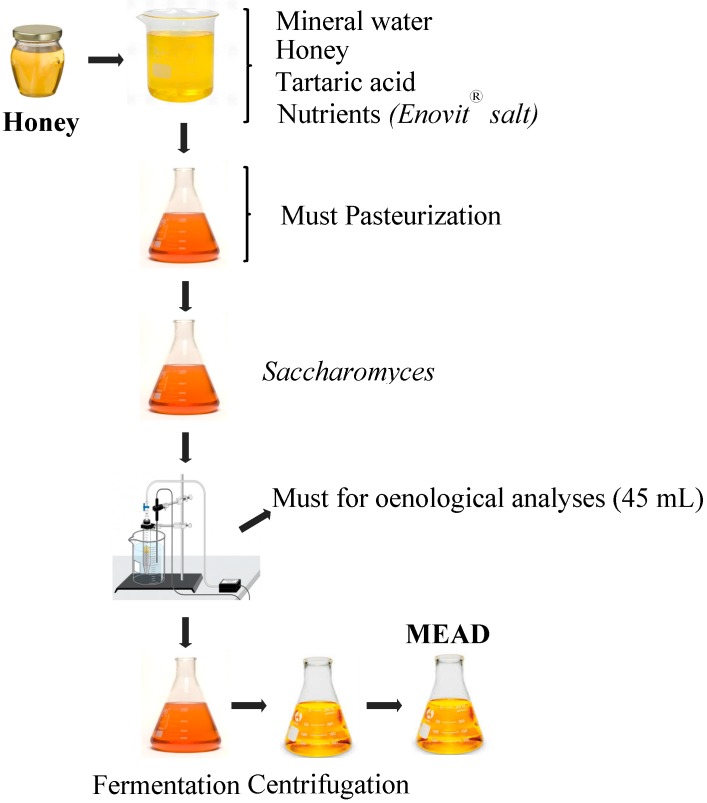
General scheme of modern mead production.

The production of mead comprises several steps (see Figure 1). In general, the first step involves must preparation followed by pH adjustment. Then, the must pasteurization, yeast inoculation, fermentation and post-fermentation take place successively. Finally, the obtained mead is centrifuged to remove undesired material.

### 4.1. Honey-Water Mixture and Additives

The must preparation consists basically in the mixture of water and honey; even though the water proportion can be replaced by fruit juice depending on the type of mead to be produced [3]. According to the Portuguese law, honey is defined as the sweet viscid material produced by bees from plant nectar, secretions of living parts of plants or excretions of plant sucking insects [28]. Afterwards, honeybees gather, transform and combine this mixture with substances of their own and store it in the honeycomb to ripen and mature.

Honey is the raw material for meads’ production consequently it greatly influences the production and characteristics of mead [7]. Its quality is influenced by the amount of microorganisms present that depends on the type of honey and water content, since low water content inhibits microbial growth. In addition, the bacteriostatic or bactericidal activity, the low pH and the high content of reducing sugars present in the honey, can prevent the growth of many microorganisms [29]. The chemical composition of this natural product is complex, including more than 180 substances, among which carbohydrates, organic acids, amino acids, proteins, minerals, vitamins, lipids, aroma compounds, flavonoids, phenolic acids, 5-hydroxymethylfurfural (HMF) pigments, waxes, pollen grains, several enzymes and other phytochemicals [30,31,32,33,34,35,36,37].

Figure 1, firstly, illustrates that the honey is diluted in water, and a mixture of nutrients is added. The proportion to which honey is diluted determines the type of mead obtained: the finest at 1:0.5 (honey:water) and the other variants at 1:1, 1:2 and 1:3. The mixtures containing the highest sugar concentrations (1:0.5 and 1:1 types) are obtained by successively adding honey, in order to avoid stopping the fermentation due to excessive osmotic pressures [27].

This may include commercially available yeast energizers [2,6,8,26]. Prior to or after fermentation, various additives such as fruit pulps or juices, citric acid and/or pollen may be also added [1,38,39]. These additives are used to speed up the fermentation, improve fermentation rates, alcohol yields and the final characteristics of meads [40,41]. Among the most common additives are: ammonium sulphate, potassium phosphate, magnesium chloride, citric acid, sodium citrate, biotin, pyridoxine, myo-inositol, calcium panthotenate, thiamine and peptone. Table 1 gathers the additives/ingredients reported by investigators from different countries.

The second step consists in the addition of a buffer, which is important to maintain the pH within a range of 3.7–4.0 throughout the fermentation [26]. Calcium carbonate, potassium carbonate, potassium bicarbonate, and tartaric acid are potential candidates. Sometimes citric or lactic acid is added (Table 1).

The next step consists in the reduction of the microbial load present in the medium to avoid the interference with the fermentation process. Therefore, in order to obtain a low load of undesirable microorganisms in the final product, the honey-must should be preferably preheated prior to the fermentation [42]. During this step, the must is subsequently sterilized, boiling being the most commonly used method [6,26,42]. However, it has been observed that heat treatments are also able to alter the antioxidant capacity of the product, by changing the phenolic profiles [5].

**Table 1:** Some additives/ingredients used in the honey-must preparation during mead production.

Country	Must Preparation	Fermentation Length (Days)	Yeast	Temp. (°C)	Ref
India	C_6_H_12_O_6_, yeast extract, peptone, MgSO_4_, ZnSO_4_ and KH_2_PO_4_	˃90	*S. cerevisiae*	18–30	[16]
Portugal	(NH_4_)_2_HPO_4_	5	*S. cerevisiae* (QA23 and ICV D47)	25	[24]
Portugal	Supplement 1: commercial nutrients *(Enovit*^®^*)* and C_4_H_6_O_6_. Supplement 2: NH_4_H_2_PO_4_; KNaC_4_H_4_O_6_·4H_2_O; MgSO_4_·7H_2_O, CaSO_4_, SO_2_, C_4_H_6_O_6_ and bentonite sodium.	8–13	*S. cerevisiae*	27	[2]
Portugal	K_2_C_4_H_4_O_6_, malic acid and (NH_4_)_2_HPO_4_	11–14	*S. cerevisiae* UCD522	25	[8]
Portugal	Commercial nutrients and SO_2_	15	*S. cerevisiae* ph.r. *bayanus* PB2002	20, 25 and 30	[23]
Slovenia	(NH_4_)_2_SO_4_ , KH_2_PO_4_ , MgCl_2_, C_6_H_8_O_7_, NaH_2_C_6_H_5_O_7_ , Vit. B_7_, Vit. B_6_, myo-inositol, Vit. B_5_, Vit. B_1_and peptone		*S. bayanus* strain R2 (Lalvin)	15	[41]
Slovak	Vitamon Ultra salt^®^		*S. cerevisiae* C11-3	25–30	[6]
Poland	(NH_4_)_2_HPO_4_ and C_6_H_8_O_7_	25–30	*S. cerevisiae*, Johannisberg-Riesling (JR)	20–22	[27]
USA	A honey analog (38% fructose, 30% glucose, 10% maltose, and 2% sucrose) diluted with H_2_O and ethanol	28–42	(*S. cerevisiae*) (LD Carlson)	22	[5]
Portugal	K_2_C_4_H_4_O_6_, malic acid and (NH_4_)_2_HPO_4_		*S. cerevisiae* Lalvin QA23 and *S. cerevisiae* Lalvin ICV D47	22	[3]
Nigeria	H_2_SO_3_ and SO_2_	21	Packaged dried bakers’ yeast	25–26	[42]
Slovak	Not additives	60–90	*Saccharomyces*	15–22	[43]
Spain	K_2_S_2_O_5_ and pollen		*S. cerevisiae*, ENSIS-LE5^®^	25	[1]

Some studies report less aggressive techniques to reduce the microbial load, including the addition of metabisulphite, either sodium or potassium salts that liberate sulphur dioxide which inhibits or eliminates the majority of microorganisms [1,26]. Also, the use of gas sulfur dioxide [2,39], of processes like pasteurization [8] and ultrafiltration [26] has also been implemented. Some of these methods have disadvantages related to the protein removing by denaturation and coagulation. After pasteurization, the must is inoculated with previously selected yeast. Results obtained in a recent study in which the fermentative performance of two yeasts was evaluated, demonstrated that increased pitching rates significantly reduced the duration of the fermentative process, even though exaggerated inoculum could decrease the production of desirable aromatic compounds [3].

After yeast inoculation, regular aseptic sampling is carried out for monitoring fermentation and growth parameters [8,24]. In the end, a filtration/centrifugation is done in order to obtain final mead [7].

### 4.2. Mead Fermentation Progress

Mead fermentation and maturing is a time-consuming process taking from weeks to several months to complete, being the quality of the final product highly variable [2,3]. Previous authors used *S. cerevisiae* cells immobilized in alginate beads to produce mead in a continuous way, achieving more than 3 months of operation [40]. The fermentation of honey solutions is known to be difficult due to their high sugar concentration derived from wort and the resultant high osmotic pressure [3] or presence of some inhibitory agents [38]. However, some places in Africa and South America produce a tropical type of honey, which is very liquid and speedy to ferment [6,27].

The progress of mead fermentation depends on several factors [3,6], highlighting the importance of the yeast strain and nutrition, pH’s control, mixing during the process [7], lack of essential nutrients such as a deficiency in available nitrogen [8] and low mineral concentration [27]. Therefore, optimal growth conditions are required in this process.

## 5. Postfermentation Conditions

Once the fermentations end, adjustments and maturation conditions are obligatory, despite the increase in production costs. For clarification, bentonite is often used [1,2,26], as well as gelatin [1].

## 6. Mead Quality Procedures

According to Kahoun *et al.*, the most informative parameters in evaluating mead quality are the hydroxymethylfurfural (HMF) and phenolic contents [44]. HMF is a cyclic aldehyde formed by the sugars degradation resulting in the reduction of the nutritional value of the product. Also, previous authors reported that this compound results from the dehydration of hexoses in acidic conditions [45], and its formation kinetics varies directly with temperature, acting as an indicator of overheating and storage in poor conditions [46].

The use of HMF as a quality index is based on the fact that, as this compound is absent in fresh honey, its final concentration in honey is only due to storage and/or heating. This is particularly important for multifloral honeys when compared with unifloral ones, since these two types of honey have a very different chemical composition [46]. The presence of HMF directly influences the color, flavors and bud odor; hence, it is used as a critical parameter of quality of honey [47]. According to the Codex Alimentarius Commission, the HMF concentration in honey should not exceed 40 mg/kg or 80 mg/kg for tropical honey. High concentrations of HMF in honey indicate overheating, poor and prolonged storage conditions or aged honey [47,48,49,50].

Regarding the assessment of mead’s quality, high concentrations of HMF and absence of most common phenolic compounds are indicators of excessive heating during the production. Also, it is very likely that some phenolic compounds can be used as indicators of mead composition and quality. Indeed, the detection of abnormally high concentrations of some compounds, for instance, vanillin, or even their presence in other cases, may be indicative of adulteration [7].

## 7. Problems Associated with the Mead Production

During the fermentative process, several problems may occur, being the most common the inability to achieve the desired alcoholic content, the existence of long and stuck fermentations and the heterogeneity of the final product [24].

In addition, yeast re-fermentations and/or bacterial secondary fermentations may occur, resulting in the production of lactic and acetic acid and increasing the production of undesirable volatile esters triggered in undesirable aroma [7,51]. According to a recent study of our work team [24], the most common undesirable compounds associated with off-flavors are ethyl acetate, octanoic acid and hexanoic acid. The combination of these compounds modifies the sensory quality of mead, specifically the aroma and flavor, making it unpleasant.

In agreement with the described by Gupta and Sharma [4] the conventional methodology used in the production of mead, which involved long heat times required for honey pasteurization, was associated with the production of off-taste, described as rubbery and resin like tastes.

Also, yeasts remaining on the product after fermentation, due to ineffective filtration procedures, can produce undesirable flavors, among which estery, acidic, phenolic or hydrogen sulphide (odor of rotten eggs) [52].

Delays and pouts during fermentation process, are others problems usually encountered in mead due to the fact that honey presents low levels of nitrogen and mineral substances, interfering in the fermentation process. According to Mendes-Ferreira *et al.*, tartaric acid helps to prevent pouts during the fermentation process [8]. Besides, the inappropriate amount of assimilable nitrogen in the fermentation can lead to poor growth of the yeast, prolonged fermentation, reduced growth rates and consequently decrease productivity. The minimum requirements of nitrogen are interconnected with the growth rate of the yeast and the concentration of ethanol [53].

The different types of honey also influence the fermentation; dark honey is richer in minerals than light honey, thus there is interference in the fermentation. Pereira *et al.* studied the ability of the yeast *S. cerevisiae* to produce mead, with honey from the region of Tras-os-Montes [2]. This author found that it is extremely important the honey characteristics (type of honey used), and supplements used for best results in the mead production. As expected, the best results were found with dark honey than with clear honey, due to that the dark honey is richer in minerals and higher pH.

Also, temperatures above 25 °C together with a higher concentration of sugars (glucose and fructose) and other nutrients increase sugars’ consumption. On the other hand, lower temperatures (less than 25 °C) and reduced nutrients’ concentrations are associated to final glucose and fructose concentrations higher than 3.5 and 10 g/L, respectively, which may promote the occurrence of undesirable re-fermentations [2]. In contrast, Šmogrovičova *et al.* reported that the low fermentation temperature helps to achieve a steady fermentation and a better transformation of the aromatic and taste qualities of the ingredients into the final product [43]. Depending on the fermentation condition and dilution of honey, mead is usually fermented for 2 to 3 months.

For *S. cerevisiae*, higher fermentation rates are obtained at temperatures between 20 and 30 °C, while temperatures lower than 15 °C are associated with significant decreases on the fermentative performance, involving consequently higher fermentation periods. However, it is important to note that the fermentation rate also decreases when temperature is above 30 °C [23]. In their extensive study, Gomes *et al.*, suggest that in order to optimize mead production, the specified targets as well as ethanol concentration could be between 11.5% to 12.3%, acetic acid 0.10 to 0.65 g/L, glycerol 6.0 to 7.0 g/L, glucose 2.5 to 3.5 g/L and fructose 5.0 to 10.0 g/L. These authors also reported that to produce mead within these limits, the optimum operational temperature is 24 °C and the nutrients concentration of 0.88 g/L (88 g/hL) [23].

## 8. Cells Immobilization

Recently, the use of immobilized cells in the fermentations has been extensively tackled by numerous investigations worldwide, as a strategy to solve the difficulties encountered during the process [54,55,56,57]. Indeed, the microorganisms’ immobilization methods have gained attention in the last few decades and are being successfully used in diverse biotechnological applications, among which the production of alcohols (ethanol, butanol and isopropanol), organic acids (malic, citric, lactic and gluconic acids) and enzymes (cellulose, amylase and lipase) [58]. Immobilized cells have also been used for the biotransformation of steroids in the wastewater treatment and in food applications, particularly beer and wine [58]. The entrapment of microorganisms in beads is commonly done by the ionotropic gelation of macromolecules with multivalent cations. The porous matrix is synthesized *in situ* around the cells, being the immobilization achieved by mixing the microorganisms with an anionic polymer, followed by cross-linking with the multivalent cations, in order to form a structure that entraps the microorganisms [57].

The advantages of systems of cell immobilization in fermentations when compared to fermentations with free cells take place in both technological and economic fields [59,60], leading to the continuous use of cell and cell protection from inhibitory substances that may be present in the medium [57]. For example, *Schizosaccharomyces pombe* cells encapsulated in beads of calcium alginate were able to reduce the fixed wine acidity, degrading malic acid [61]. Quality tests conducted with alginate beads showed that the immobilized cells could be recycled up to five times without the liberation of the cells into the wine must [61]. Bezbradica *et al.* studied the immobilization of beer yeasts in polyvinyl alcohol and observed a high fermentation rate with an amount of 10^9^ cells/mL, a reduced fermentation time and a high mechanical stability: thirty days of fermentation in operation for six months without significantly altering cellular activity [62].

However, Genisheva *et al.*, conducted a study with immobilized yeasts, which reduced the fermentation time and the concentration of SO_2_, with a higher concentration of ethanol [59]. Meanwhile, Pereira *et al.*, in recent studies, evaluated the effect of using immobilized cell systems on mead production [24]. The obtained results demonstrate that the immobilization of yeasts in Ca-alginate did not affect negatively the fermentation process. Minor differences were detected in the fermentation length and in the rate between fermentations conducted with free or immobilized cells, even though higher concentrations of viable cells were achieved in immobilized systems. The results of the fermentation kinetics profiles of the free or immobilized cells, expressed in terms of sugar consumption, showed that, in all fermentations, 50% or more of the sugars were consumed after 48 h of fermentation. Nevertheless, fermentations conducted with different systems reached the same final ethanol concentration, 10% to 11% vol. Similar studies have been reported higher productivity in the immobilized system in comparison of the free cell system [63]. However, it is important to state that the fermentation productivity depends on the concentration of yeast cells immobilized in beads, on the bead-size, as well as on the temperature of fermentation [60]. Although the most aromatic meads were the ones produced by immobilized cells, the odor activity values of undesirable compounds were also higher in these fermentations. As such, it appears that immobilization has minor advantages for mead production [24]. Other studies with immobilized cells in Ca-alginate [22] or pectate [6] in mead production have showed that fermentation length was reduced or fermentation rate increased, respectively. Immobilized cells presented an increased energetic metabolism, storage of polysaccharides, substrate uptake, increased product yield and reduced formation of by-products, higher values of intracellular pH, increased tolerance against inhibitory and toxic compounds and higher invertase activity [64].

However, all these advantages are dependent on the stability of the immobilization matrix; it should be inert, insoluble, non-biodegradable and mechanically stable during the different operations in a bioreactor. In turn, mechanical stability depends on the viscoelastic properties and the concentration of sodium alginate solution [6,65].

## 9. Conclusions

Mead is the first alcoholic beverage known to man, which results from the fermentation of a honey solution carried out by adequate yeasts. Particularly when produced in a traditional way, the fermentative process may be complicated by several problems, among which delayed or arrested fermentations, development of unpleasant aromas and production of meads with low quality. These are commonly due to the stressful and unfavorable growth conditions to which yeasts have to respond and adapt.

Cell immobilization is currently a key strategy to overcome these inconvenient and recent studies evidence its exceptional advantages when compared to free cells. Immobilized cells have shown diverse possibilities to facilitate the accomplishment of fermentation, not only on mead production but also in the field of sparkling wines production. Furthermore, enhanced productivity, greater tolerance to inhibitors and elimination of contaminants can occur. The main shortcoming of immobilized cells implementation on industrial scale is that a specialized personal and a strong scientific understanding about the effect of immobilization on physiology of industrial strains are required. Considering that the sensorial attributes of mead are increasingly appreciated, studies on the production of this alcoholic drink at low costs should be undertaken. A viable hypothesis would be to obtain mead by using honeys of second category, providing added value to beehive products, economic benefits to the beekeepers and differentiated good quality products to the consumers.
